# Functional and Therapeutic Relevance of Rho GTPases in Innate Immune Cell Migration and Function during Inflammation: An *In Silico* Perspective

**DOI:** 10.1155/2021/6655412

**Published:** 2021-02-13

**Authors:** Pankaj Dipankar, Puneet Kumar, Shiba Prasad Dash, Pranita P. Sarangi

**Affiliations:** Department of Biotechnology, Indian Institute of Technology Roorkee, Roorkee, Uttarakhand 247667, India

## Abstract

Systematic regulation of leukocyte migration to the site of infection is a vital step during immunological responses. Improper migration and localization of immune cells could be associated with disease pathology as seen in systemic inflammation. Rho GTPases act as molecular switches during inflammatory cell migration by cycling between Rho-GDP (inactive) to Rho-GTP (active) forms and play an essential role in the precise regulation of actin cytoskeletal dynamics as well as other immunological functions of leukocytes. Available reports suggest that the dysregulation of Rho GTPase signaling is associated with various inflammatory diseases ranging from mild to life-threatening conditions. Therefore, it is crucial to understand the step-by-step activation and inactivation of GTPases and the functioning of different Guanine Nucleotide Exchange Factors (GEFs) and GTPase-Activating Proteins (GAPs) that regulate the conversion of GDP to GTP and GTP to GDP exchange reactions, respectively. Here, we describe the molecular organization and activation of various domains of crucial elements associated with the activation of Rho GTPases using solved PDB structures. We will also present the latest evidence available on the relevance of Rho GTPases in the migration and function of innate immune cells during inflammation. This knowledge will help scientists design promising drug candidates against the Rho-GTPase-centric regulatory molecules regulating inflammatory cell migration.

## 1. Introduction

**Figure 1:**
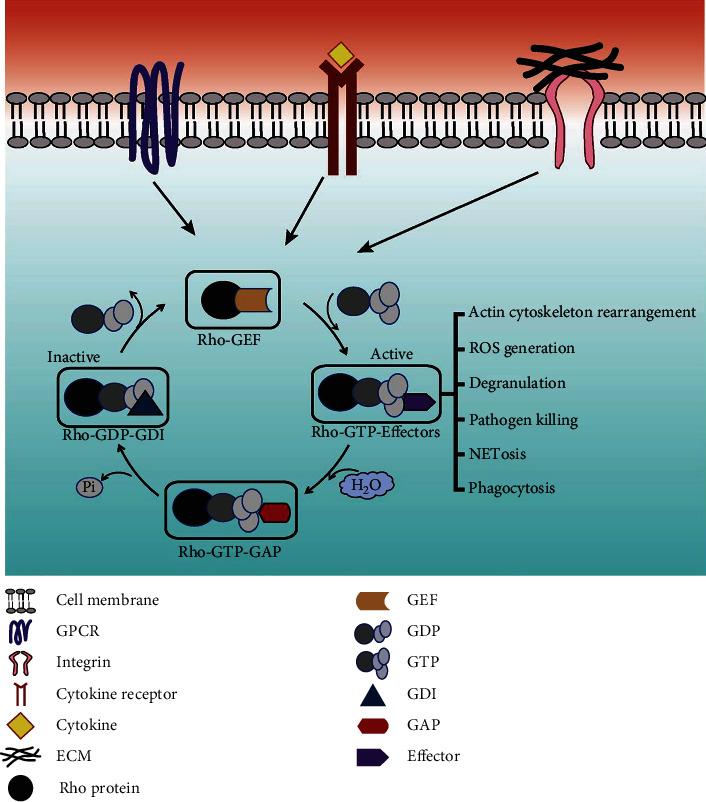
Overview of activation and regulation of Rho proteins during inflammation. Extracellular signals mediated through cell surface receptors such as GPCR, integrins, and cytokine receptors lead to the activation of RhoGTPases, resulting in various effector functions of inflammatory immune cells. The precise regulation of Rho proteins is performed by mostly three regulatory proteins, namely, GEFs, GAPs, and GDIs. Rho proteins primarily active in GTP-bound form become nonfunctional in GDP-bound form. Guanine Nucleotide Exchange Factors (GEFs) activate the Rho proteins by exchanging GDP for GTP, and in GTP-activated form, GTPases bind to different effectors and perform a downstream cellular function such as actin cytoskeleton rearrangement, cell cycle progression, and gene expression. GTPase-Activating Proteins (GAPs) enhance the intrinsic GTP hydrolysis of Rho-GTPase by releasing inorganic phosphate (Pi), thereby inactivating GTPases. The third regulatory protein is guanine nucleotide dissociation inhibitors (GDIs), which keep Rho proteins in GDP-bound form and prevent the localization of GTPases from the cytosol to the plasma membrane, and protects them from the action of GEFs.

Inflammation is a biological response against microbial infections and tissue injury that involves the infiltration of immune cells and the release of soluble inflammatory mediators leading to vascular changes [1]. During inflammatory responses, the recruitment of immune cells to the site of inflammation is mediated by several cell adhesion receptors and signaling molecules, including Rho GTPases, that coordinate the firm adhesion, elongation, and protrusion formation processes in response to signals received from various molecules such as chemokines [2]. The mammalian Rho GTPases belong to the Ras superfamily of small GTPases, which is encoded by more than 20 genes, including RhoA, RhoB, RhoC, Rac1, Rac2, Rac3, Cdc42, TC10, TCL, RhoD, RhoG, RhoE/Rnd3, Rnd1, Rnd2, Chp1, Chp2, RhoBTB1, RhoBTB2, Rif, and TTF [3]. From the aforementioned members, most of the studies have focused on the roles of Cdc42, RhoA, and Rac1 in cellular processes during physiological and pathological conditions compared to other Rho proteins [3, 4]. Studies show that in various immune cell types, RhoA induces the formation of stress fibers and Rac1 induces the extension of actin-based protrusions called lamellipodia. In contrast, Cdc42 induces the extension of finger-like plasma membrane protrusions known as filopodia, which are further driven outward through actin polymerization [3]. In addition to the regulation of cytoskeletal machinery and cell migration, the above GTPases are also shown to modulate inflammatory functions such as Reactive Oxygen Species (ROS) generation, degranulation, pathogen killing, NETosis, and phagocytosis in immune cells like neutrophils and macrophages (as shown in Figure 1) [5, 6]. Additionally, Rho GTPases are involved in hematopoiesis and leukocyte development [7, 8].

The classical Rho proteins are in OFF state when it binds with GDP, while ON state refers to the binding with GTP [9]. The active form or GTP-bound Rho proteins bind with different downstream effector proteins (GTP has 100-fold higher affinity than GDP to bind with effector proteins) [10–12] and modulate different signaling pathways to perform a distinct cellular function such as adhesion, migration, phagocytosis, cytokinesis, cellular morphogenesis and polarization, growth, and cell survival [13–16]. Therefore, in this way, cycling between ON/OFF state Rho proteins act as a bimolecular switch and control different cell signaling pathways (Figure 1). During the activation of Rho GTPases, Mg^+2^ acts as a cofactor for both GTP and GDP bindings. Moreover, small Rho proteins possess different conserved domains for nucleotide binding, GTPase activity, and effector protein binding sites [12]. The cycling between GDP and GTP-bound states is primarily mediated by two regulatory proteins including GEFs (Guanine Nucleotide Exchange Factors) by exchanging GDP to GTP form by displacing the Mg^+2^ and initiating the switch from GDP to GTP state. Simultaneously, inactivation is catalyzed by GAPs (GTPase-Activating Proteins), which mainly convert GTP into GDP state by hydrolyzing GTP. Thus, GAP suppresses the activity, but GEF enhances the activity of Rho proteins by the interaction of the effector binding interface acting as switch I and switch II of Rho proteins [17, 18].

Evidence from multiple studies has shown that a defect in immune cell migration and localization could result in the development of the severe form of inflammatory diseases such as systemic lupus erythematosus (SLE), rheumatoid arthritis (RA), Crohn's disease, chronic obstructive pulmonary disease (COPD) and ulcerative colitis which is characterized by cytokine storm, and unresolved inflammation. In this review, in addition to the discussion about the role of Rho GTPases (e.g., Cdc42, RhoA, and Rac1) in the neutrophil and macrophage migration and functions during the inflammatory responses, we will also highlight various domains of these molecules that could be targeted for therapeutic purpose in multiple diseases. [7, 19]. Thus, a precise modulation and selective targeting of innate immune trafficking have been proposed as an effective therapeutic in many inflammatory disease pathologies [19, 20]. As mentioned above, Rho GTPases act as molecular switches for immune cell migration events, making them an important therapeutic target for modulating immune cell migration and function and alleviating inflammation-associated diseases. In this review, we have summarized the latest findings on the role of the critical Rho GTPases (RhoA, Cdc42, and Rac1) in the migration and function of innate immune cells. We have provided information on the structural aspects of these molecules' different domains with their relevance in designing targeted therapeutics.

## 2. Role of GTPases in Innate Immune Cell Migration and Function

Innate immune cells such as neutrophils, monocytes/macrophages, and dendritic cells play an essential role in acute and chronic inflammatory conditions [19]. Several studies have highlighted the role of RhoA, Cdc42, and Rac1 in the regulation of the recruitment and functional events of neutrophils and monocytes/macrophages primarily by controlling their actin and tubulin cytoskeleton rearrangements [7].

In neutrophils, their migratory behavior under inflammatory conditions is mediated by either chemoattractants and/or shear stress. Similarly, chemokines are also shown to mediate cell polarization processes (e.g., bulging pseudopod formation) in these cells [21]. This well-organized activity is regulated via F-actin formation through Rac GTPase activation and withdrawing uropod controlled by functional RhoA. Neutrophil migration due to shear stress is thought to involve the instigation of RhoA specific GEF-H1 [22]. A recent study showed the role of Rho GTPases in NETosis during sepsis. In the study, using the CLP model of murine sepsis, the extracellular CIRP and TREM-1 axis was shown to increase ICAM-1 expression and Rho activation, leading to increased NETosis, which further exacerbates the inflammation [6]. RhoA activation also has been shown to be essential for TLR-2 and TLR-4 mediated inflammatory cytokine production by human monocytes [23, 24]. In guided migration activity, active RhoA is well known for its familiar role in managing the cell's tail extremity withdrawal. Koenigs et al., in their work, showed that genetically modified mice deficient in Rho GTPase macrophages suffered an extended tail part due to failure in uropod retraction [25]. Under inflammatory conditions, proinflammatory (TNF*α* signaling via PI3K and PKC-*ζ*) and anti-inflammatory TGF*β* (macrophage inflammatory protein-1*α* induced RhoA) cytokines are shown to differentially modulate the directional migration of macrophages via activation of RhoA. Directional migration is also mediated via local reduction of RhoA in podosomes which contains matrix-degrading enzymes via GEF, PAK1, and ARHG7 (Rho/Rac-specific GEF) [26]. The roles of RhoA in various innate immune cells are reviewed in the article by Bros et al. [27].

Studies in human (HL-60) and murine neutrophils have demonstrated that Cdc42 plays an essential role in regulating actin and tubulin organization, cell-matrix interactions, and maintenance of cell polarity [28]. The directionality quotient in cell migration in neutrophils is associated with RhoA and Cdc42, where they control the spatiotemporal behavior of PTEN, which further influences the PI3K behavior needed for direction [29]. Several complex mechanisms are responsible for the dual obstruction of RhoA and Rac required for neutrophil migration. The phosphorylation of the Rac1-specific GAP FilGAP is mediated by ROCK stimulation through RhoA, and this FilGAP attaches to filamin A situated at the cell face lowering the Rac [29]. Moreover, a recent study showed the involvement of Cdc42 in random and directed migration, activation, degranulation, and formation of Reactive Oxygen Species (ROS) and in the regulation of pathogen killing efficiency by neutrophils. These Cdc42-regulated neutrophil effector functions were attributed to the differential regulation of Akt, p38, and p42/44 [5]. Similarly, Weber et al. showed the role of Cdc42 in chemokine-induced monocyte transmigration. The signaling pathway involved in the process was mediated by PI3K in the upstream, resulting in Cdc42-mediated cytoskeletal rearrangement [30]. In human alveolar macrophages, Cdc42 and RhoB activation is essential for mannose receptor-mediated phagocytosis [31]. Cdc42 also acts as a critical regulator in multilineage blood development, where it regulates the balance between erythropoiesis and myelopoiesis. The deletion of Cdc42 leads to a decrease in erythropoiesis and an increase in myelopoiesis, which are linked to the downregulated proerythroid genes and upregulated promyeloid genes [8].

Similarly, Rho GTPase Rac1, expressed in neutrophils and macrophages, was shown to mediate the recruitment of neutrophils to inflammatory tissues (e.g., lungs) and associated with the regulation of the morphology of macrophages and macrophage migration [32, 33]. In the aforementioned studies, Rac-1 was shown to mediate the actin cytoskeleton rearrangement [33]. In support, a separate study demonstrated that Rac1 null macrophages demonstrated a defect in cell spreading and ruffle formation around membranes [34]. Arbibe et al. showed the requirement of Rac1 activation in the TLR2-mediated NF-*κ*B (a critical molecule involved in the inflammatory response) activation in THP1, a human monocytic cell line [35]. Like Rac1, Rac2 (another isoform of Rac1) was also shown to be essential for regulating phagocytosis, superoxide production, and recruitment to the site of inflammation and required for normal morphology of macrophages [33].

Thus, the presented evidence indicates that RhoA, Cdc42, and Rac1 are essential for the regulation of macrophage and neutrophil migration and function. Therefore, a better understanding of the structural organization of various functionally active domains will help in designing novel Rho GTPase centric therapeutic molecules.

## 3. Rho GTPase Activation and Functional Correlation with Key Structural Domains

### 3.1. Mechanism of Activation

As explained above and shown in Figure 1, Rho protein activation is often mediated by various cell surface receptors, such as adhesion receptors, cytokine receptors, tyrosine kinases, and G-protein-coupled receptors (GPCRs). Like Ras, Rho protein (a monomeric G-protein), acts as a molecular switch in response to GDP and GTP binding and adopts a distinct conformation and activates downstream signaling [36, 37]. The GDIs, GEFs, and GAPs are the central controllers in the activation sequence of GTPases. GDP-bound GTPases are well insulated by GDIs, thus avoiding the GDP detachment from GTPases. GDIs ensure blocking any interactions with target proteins and regulatory molecules and help sustain GTPases in a soluble cytoplasmic state. Then, comes the GEFs that help catalyze the detachment of attached GDP, thereby promoting the association of GTP with GTPase. Additionally, the activated GTPase-mediated downstream signaling is guided by GEFs via the creation of a precise GTPase-GEF-effector molecule complex and a unique arrangement of spatially controlled cell stimulation. Finally, the GAPs encourage an inherent GTP hydrolytic behavior of the GTPases, which subsequently bring back the active state (GTP-bound form) to the inactive state (GDP-bound form) [9].

The mammalian Rho GTPases belong to the Ras superfamily of small GTPases encoded by more than 20 genes. The classical Rho GTPases such as RhoA (RHOA, RhoB, and RhoC), Cdc42 (comprising of CDC42, RHOJ, and RHOQ), Rac subfamily (including RAC1, RAC2, RAC3, and RHOG), and RhoF (consist of RHOD and RHOF) are regulated by GDP/GTP exchange [38]. Additionally, activation of these Rho GTPase is also regulated by other mechanisms including posttranslational modifications (PTMs) such as phosphorylation, ubiquitylation, sumoylation, and regulation of Rho GTPase mRNA at the gene expression or posttranscriptional level by miRNAs [9]. For example, RhoA expression is regulated by miR-33, miR-133, miR-155, and miR-185. Cdc42 expression is regulated by miR-1, miR-29, miR-124, miR-133, miR-137, and miR-185. The expression of Rac1 is regulated by miR-124 [39]. Atypical Rho GTPases are regulated through lipid-based posttranslational modifications at their C-terminus.

Interestingly, the GTP to GDP-bound form and vice versa are regulated by more than 80 RhoGEFs and 69 RhoGAPs that mediate the activation process under various physiological and pathological conditions [40]. However, our understanding on how specific signals guide particular Rho GTPases and activate specific cellular signaling pathways is still limited. The regulation of Rho proteins is complicated, and it raises so many unsolved questions like how the activity of Rho proteins is spatiotemporally regulated in the different immune cells and the criteria for selectivity and specificity of GEFs and GAPs are not well understood. Furthermore, their role in the regulation of cytoskeleton rearrangement during migration needs to be addressed.

### 3.2. Structural Basis of Rho GTPase Signaling

#### 3.2.1. Overall Fold and Conformation of Rho GTPases

**Figure 2:**
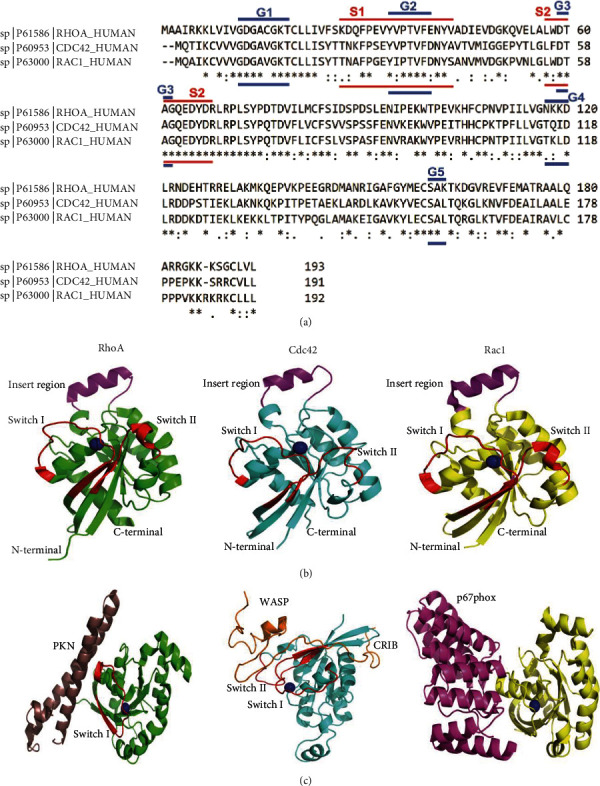
Sequence alignment and structural representation of RhoA, Cdc42, and Rac1. (a) FASTA sequence of RhoA, Cdc42, and Rac1 was retrieved from the Uniprot database, and sequence alignment was performed through the Clustal Omega tool. The length of different G-boxes (G1, G2, G3, G4, and G5) in the G domain is marked in the blue line, while the length of switch regions is highlighted in the red line. Asterisk represents the entirely conserved column; colon indicates the column where all residues have approximately the same size and hydropathy; full stop indicates the column where the size or hydropathy has been preserved in the course of evolution. (b) Structural representation of RhoA (green color, PDB: 1CXZ), Cdc42 (cyan color, PDB: 1CEE), and Rac1 (yellow color, PDB: 1E96) with their switch I and switch II region highlighted in red and insert region highlighted in magenta color. (c) Structural depiction of RhoA-PKN complex (RhoA in green color and PKN in light brown, PDB: 1CXZ), Cdc42-WASP complex (Cdc42 in cyan color and WASP in orange color, PDB: 1CEE), and Rac-p67phox complex (Rac1 in yellow color and p67phox in bright pink color, PDB: 1E96). The blue color sphere represents the Mg^2+^ ion.

Innate immune cells play a very vital role in inflammatory processes. Any aberration in the regulation of their recruitment could significantly change the outcome of any inflammatory response. Evidence from the literature supports that the 3-dimensional (3D) structure of proteins plays a decisive part in regulating the fate of any cellular signaling pathway [41]. Thus, to decipher the complex regulation of GTPase functions, a closer investigation of their structural aspects is essential. The intrinsic behavior of Rho GTPases relies on the Mg^2+^ ion-dependent state of guanine nucleotide binding conformations. A signaling cascade is initiated when Rho GTPases in their active state binds to a specific group of proteins (i.e., effectors). The functional domains in Rho GTPases are very much similar to the Ras. As shown in Figure 2(a), there are four sections in the functional domain that participate in the attachment and hydrolysis of guanine nucleotides, namely, G1, G3, G4, and G5 boxes [42]. The G2 box is involved in the communication with the effector molecules, and a terminal CAAX box is also present. The importance of CAAX (C = cysteine residue, A = aliphatic residue, and X = any other residue) box lies in the fact that it acts as an indicator for protein prenylation through protein prenyltransferases [43]. The switch regions take the center of attraction in the active and inactive state of Rho GTPases. Ihara et al. showed the structural differences between GDP and GTP bound states of human RhoA are mainly restricted to switch I (position 28 to 44) and switch II (position 62 to 69) [44]. Similarly, the GDP- and GTP-bound conformation in Rac1 lies in switch I (positions 25 to 49) and switch II (positions 59 to 76). The sequence alignment along with the structural representation of G domain boxes, switch regions and insert region for RhoA, Cdc42, and Rac1 are shown in Figures 2(a) and 2(b).

#### 3.2.2. Rho Effector Identification

There are multiple effectors like Rho kinase (ROCK), protein kinase N (PKN), and rhotekin that are known to connect with Rho GTPases in GTP-governed fashion. A coiled-coil motif that is common to these effector molecules mediates the proper binding with RhoA. Closely observing the Rho-PKN (PDB: 1CXZ) structure uncovers an antiparallel coiled-coil (ACC) finger fold on the PKN domain that straightforwardly interacts with the switch I segment, beta-strand, and alpha-helix of RhoA [45]. This peculiar characteristic of RhoA helps it to stand out from the crowd of different GTPase families and Rho GTPases.

Similarly, the effectors that interact with the Cdc42 and Rac have a generic stretch of a 15-residue long motif called CRIB, aka Cdc42/Rac interactive binding motif. The reason for the common binding motif for Cdc42 and Rac lies in the fact that both have comparatively superior sequence identity (around 70%), while RhoA shares around 45% sequence identity with Cdc42/Rac. The Cdc42/Rac effectors (ACK, PAK, WASP, etc.) have CRIB motif to interact through an intermolecular beta-sheet of Cdc42/Rac and also create connection with the switch I, II, and alpha-helices [46]. The interaction with the switch I, beta-strand, and alpha-helix plays an essential factor when it comes to differentiating between Cdc42 and Rac. The p67^phox^ is an exception in this case, which is an effector molecule for Rac but lacks the CRIB motif. The structural depiction of effectors (PKN, WASP, and p67^phox^) has been shown in Figure 2(c).

#### 3.2.3. Regulator RhoGEF

RhoGEFs are multitalented and the smartest proteins in the Rho GTPase-mediated signaling. Its smartness comes from its numerous domains (e.g., more than 10 domains in Trio), thereby empowering them to perceive precise indications from upstream proteins and further activating Rho GTPases. Domain analysis of RhoGEFs suggests the presence of pleckstrin homology (pH) and dbl homology (DH) domains, and this dual component holds the least requirements for the entire GEF motion [47]. Based on the structural probe, Snyder et al. and Rossman et al. reported a preserved technique for nucleotide interchange by RhoGEFs in catalyzing Rho GTPases [48, 49]. With three extremely conserved areas, the DH domain facilitates two vital things: the first is reforming the switch segments and disrupting Mg^2+^ ion of related GTPases, and the second is nucleotide binding. The other imperative pH domain explicitly controls trade without phospholipid binding, although it is generally associated with membrane localization through phospholipid binding.

#### 3.2.4. Regulator RhoGAP

When it comes to GTP hydrolysis, the GAP is known to increase this process over many folds (up to 10^5^). The structural insight suggests that all GAP domains are bent into all alpha helix form [50]. For example, p50RhoGAP entails nine alpha-helixes and attaches to Rho GTPases via stabilizing switch I and switch II region. In the RhoGAP-RhoA complex with GDP and AlF_4_^−^ GAP tends to stabilize the intermediate position by appending an arginine finger in the active site of GTPase. A well-preserved Arg residue intermingles straightforwardly with the Gln residue at the 61^st^ position of GTPase, and this interaction favours the proper phosphoryl movement through hydrolytic water. The overall collaboration between the residues results in lowering the energy requirement for GTP hydrolysis. The highly conserved Asn residue at the 194^th^ position in p50RhoGAP also helps in the stabilization of the effector loop via primary chain interaction [46].

#### 3.2.5. Regulator RhoGDI

A well-preserved CAAX sequence stretch is present at the C-terminal of Rho GTPases, which is carefully maintained by GDIs via safeguarding it from the aqueous milieu where GDI-GTPase complexes comprise a cytoplasmic collection of prenylated proteins. The GDI-Rho GTPase complex structure investigation reveals that GDI's N-terminal region contains nine beta-strands with a short helix trailed by helix-loop-helix motif arrangement folded into an immunoglobulin-like sandwich [51, 52]. The conserved hydrophobic residues present in the hydrophobic pocket of the immunoglobulin-like domain make complementary van der Waals interactions with the geranylgeranyl chain. The switch II region predominantly forms an association with GDI and the C-terminal of the switch I region. The helix-loop-helix part of GDI has a conserved Asp residue, which makes a hydrogen bond with the switch I region of Rho GTPase. The switch II region is also in extensive connections with the GDI. With the switch I region being stabilized, it hampers the GDP separation and hydrolysis of GTP by Rho GTPase [46].

## 4. Rho GTPases as Therapeutic Targets during Inflammation

Aberrant inflammatory immune cell migration and function are associated with the severity of both local and systemic inflammatory conditions. A study by Lerman et al. showed exacerbated infiltration of hyperinflammatory neutrophils in murine and human sepsis [53]. Similarly, several pieces of evidence suggest dysregulated inflammatory immune cell functions such as migration, cytokine production, ROS production, and NETosis drive the severity of inflammatory conditions including RA, SLE, COPD, ulcerative colitis, and sepsis [19, 54]. As discussed in this review, RhoGTPases such as Cdc42, Rac1, and RhoA are involved in several effector functions of inflammatory immune cells like neutrophils, monocytes, and macrophages. Hence, targeting the RhoGTPases or upstream molecules such as integrins or integrin-ECM interaction and tyrosine kinases may be helpful in devising therapeutic strategies for inflammatory diseases. The pattern of ECM expression changes in sepsis, as recently studied by Bhan et al., may participate in the dysregulated immune cell activities involving Rho GTPases through ECM and its receptor interaction [55]. The specific interactions among different proteins direct a broad series of biological mechanisms and signaling pathways. Any irregularity in the signaling pathway might result due to the mutation over-, under-, or no production of specific proteins. Over the years, a key strategy for targeting Rho GTPases was to unsettle the Rho GTPases-GEF interactions [56].

### 4.1. RhoA

In a computational study performed by Shang et al., the *Rhosin* inhibits RhoA and its GEF LARG interaction. *Rhosin* acts by preventing RhoA activation and downstream processes like filamentous actin creation and focal adhesion association [57]. Another reported RhoA inhibitor is *Y16*, which identifies the opposite sides of RhoA and LARG to that of Rhosin. The *Y16* fails to deliver the expected results single handedly, but performs synergistically very well with *Rhosin* in a breast cancer model [58]. A unique compound known as *CHS111* helps to constrain neutrophil migration when used in a dose-dependent style and obstruct the stimulation of GEF Vav [59]. In another experiment by Castoreno et al., *Rhodblocks* 1, 3, and 6 were reported to be effective Rho pathway inhibitors without any clear inhibitory action mechanism. *CCG1423* has been tested in mouse models for inhibiting aggressive migration of prostate cancer; however, its efficacy to hinder immune cell migration is not understood [60].

### 4.2. Cdc42

*Secramine* is the first Cdc42 selective inhibitor. It makes the interaction between Cdc42 and GDIs stronger, thereby preventing the activation of Cdc42. In the in vitro experiment, *Secramine* has shown a decreased pattern of cell spreading [61]. *Ml141* (CID29950007) and its analogue CID44216842 specifically act on Cdc42 by inhibiting the nucleotide attachment. Hong et al. also emphasised the possible effectiveness of *Ml141* in immune cell trafficking [62]. The virtual screening technique gave another small molecule inhibitor of Cdc42, i.e., *ZCL278*. It is tested on various in vitro models (migration and filopodia formation) and acts by obstructing the Cdc42-GEF binding [63].

### 4.3. Rac1

*NSC23766* (a Rac1 inhibitor) is used for the inhibition of lamellipodia formation and mobilization of haematopoietic progenitor cells. It was shown to disturb the interaction between Rac1 and its GEFs (Tiam1 and Trio) without altering the interactions between RhoA or Cdc42 with their GEFs [64]. Similarly, *EHop-16*, an improved version over NSC23766, shows a dual inhibitory nature by disrupting Rac1 and Cdc42 interaction with GEF Vav1. The *in vitro* experiment performed by Montalvo-Ortiz et al. also proved the inhibition of lamellipodia formation and directed migration by EHop-16 [65]. EHop-16 was developed from NSC23766 by altering the side chains attached to the central pyrimidine ring. *Eht1864* is another noncompetitive inhibitor specific to Rac1 that disrupts the joined nucleotide, thereby arresting it in the inactive state. Rac1-mediated lamellipodia development is shown to be inhibited by Eht1864 in NIH-3T3 cells [66].

The second strategy used by researchers was to alter the actions pertaining to the upstream regulators or downstream effectors [56]. However, the presence of multiple domains in GEFs and GAPs makes it a tricky job as these multifunctional molecules have other vital cellular activities and are not confined to only Rho GTPase signaling [56]. A clinically proven drug, *Fasudil*, is one of the established inhibitors of serine-threonine kinases, including ROCK also. It makes a strong association with the ATP binding site of ROCK, thus inhibiting ATP attachment in the groove situated between N-terminal helical domain and the bilobed kinase domain of ROCK. The *in vivo* experiments of Fasudil demonstrated its interfering behavior on leukocyte recruitment, where another compound Y-27632 was studied in inflammatory diseases [67, 68]. PAK1 is also a choice of inhibition target, but due to its excessive toxicity, low specificity, and chemical variability with ATP competitive and noncompetitive inhibitors, it could never appear in the limelight.

## 5. Conclusion

The Rho GTPases play a central role in the immune cell migration process. Still, dysregulation of Rho-GTPases results in various diseases characterized by abnormal cytoskeletal dynamics, such as developmental defects, immunodeficiencies, and tumor metastasis. Therefore, Rho-GTPase-related regulatory machinery such as GEFs and GAPs and specifically binding effector proteins emerged as desirable pharmacological targets, particularly in inflammatory disorders where blocking tissue infiltration by leukocytes can be both prophylactic and therapeutic. Recent studies showed reduced infiltration of inflammatory neutrophils and macrophages and improved survival in murine sepsis by targeting integrins using C-terminal fragments of an extracellular matrix protein fibulin 7 [69, 70]. Although the involvement of RhoGTPases was not investigated in these studies, Fbln7 is known to be involved in regulating RhoGTPase signaling and related cellular activities [71]. To this end, publicly accessible databases like NCBI and OMIM contain the mutational information occurring in human Rho GTPases. Logical filtering of these mutational data and narrowing it down to the most damaging mutation will help to comprehend the structural-functional relationship of Rho GTPases in inflammatory conditions. This will enable different biomedical applications like drug design and remedial involvement to target immune cell migration. *In silico* identification of small inhibitors for overproduced Rho GTPases in inflammatory scenarios requires structural investigation of the involved proteins. Although a lot of Rho GTPases have been structurally solved and deposited in RCSB PDB, many of Rho GTPase structures are still not resolved. Computational approaches like protein modeling could be taken into consideration to make a relatable correlation among different Rho GTPases. Structural models would facilitate the recognition of an allosteric binding site on the protein surface that could be aimed at inhibiting the protein. These binding sites could be targeted by small inhibitors either from chemical libraries like ZINC database or from natural compound databases like CMAUP (Collective Molecular Activities of Useful Plants). Screening the most potent compounds from these databases through virtual screening techniques, validating through molecular docking, and further looking into protein-ligand stability through molecular dynamics simulation will help to explore new or better alternatives of the currently available medicines for Rho GTPases.
